# Response of leaf biomass, leaf and soil C:N:P stoichiometry characteristics to different site conditions and forest ages: a case of *Pinus tabuliformis* plantations in the temperate mountainous area of China

**DOI:** 10.3389/fpls.2022.1060406

**Published:** 2022-12-20

**Authors:** Yutao Wang, Yiming Zhang, Lijiao Wang, Xin Jing, Lei Yu, Ping Liu

**Affiliations:** ^1^ College of Forestry, Shenyang Agricultural University, Shenyang, China; ^2^ Key Laboratory of Tree Genetics, Breeding and Cultivation in Liaoning Province, Shenyang Agricultural University, Shenyang, China; ^3^ Engineering Technology Research Center of Chinese Pine of National Forestry and Grassland Administration, Beijing Forestry University, Beijing, China

**Keywords:** ecological stoichiometry, *Pinus tabuliformis* plantation, forest ages, site conditions, biomass of the leaves

## Abstract

Ecological stoichiometry is an important index that reflects the element cycle and ecosystem stability. In this study, two sites (sunny and shady slopes) and five forest ages (young stage, half-mature stage, near-mature stage, mature stage, and over-mature stage) in a *Pinus tabuliformis* plantation were chosen to illustrate the effects of forest ages and site conditions on the biomass and stoichiometric characteristics of leaves and soils in the temperate mountainous area of China. Except for young stage, the biomass of the leaves of *P. tabuliformis* on sunny slopes were higher than those on shady slopes in other forest ages, the average carbon content of the leaves in sunny slopes was higher than that in shady slope, while the average total nitrogen contents and average total phosphorus contents of the leaves showed the opposite of this. The biomass of leaves increased on sunny slopes, and increased first and then decreased in shady slopes with increasing forest ages. The contents of soil total carbon (STC) and soil total nitrogen (STN) decreased with increasing soil depth, while the soil total phosphorus (STP) and soil available phosphorus (SAP) contents displayed the opposite. In addition to SAP, the average content of STC, STN, and STP in shady slopes was higher than that in sunny slopes, and the ratio was the opposite. Except for STC: STN on shady slopes, the other ratios showed a downward trend with an increase in soil depth. Excluding the topsoil, the change trend of STC : STP and STN : STP in shady slopes and sunny slopes was consistent with forest ages. The results showed that forest ages and site conditions had significant effects on leaf biomass. The biomass of the leaves is mainly limited by nitrogen. These results have important significance in improving the refinement of local forestry management of *Pinus tabuliformis* plantations in the temperate mountainous area of China.

## 1 Introduction

Forest ecosystems are the main constitute of terrestrial ecosystems (Wang et al., 2008), which play a pivotal role in coping with global climate change, maintaining ecological balance, and protecting biodiversity. In complex forest ecosystems, soil is the basis for plant survival. It provides nutrients and water for plants, and plants return nutrients to the soil through dead branches and leaves. These factors are closely linked and there is an extremely close relationship between plant and soil C, N, and P stoichiometric characteristics. Based on the principles and methods of ecological stoichiometry, it is of great significance to study the ecological stoichiometry characteristics of total carbon, total nitrogen, and total phosphorus of plants and soils in forest ecosystems to reveal the nutrient limitation and the mechanism of element cycling and balance. Ecological stoichiometry is the application of stoichiometry in ecology. It is a new subject developed in the last 20 years and a new method to study plant-soil interactions and the carbon, nitrogen, and phosphorus cycle (Yang, 2020), which provides a good index and research direction for the study of plant-environment interactions (Deng et al., 2020). In ecological stoichiometry, each element has a different role, among which carbon plays the part of a structural element in the plant body, and nitrogen and phosphorus play an important role in determining the limiting factors of plant productivity (Wassen et al., 1995; Zhang et al., 2004). Studies have found significant differences in nitrogen and phosphorus contents between plant phylogenetic stages (coniferous and broad-leaved) and plant functional groups (tree, shrub, and grass), and the seasonal variation has been found to be strongly reflected in the leaves (Yan et al., 2021). The C: N and C:P ratios can represent the nitrogen and phosphorus nutrient utilization efficiency and carbon assimilation capacity of plants, and the N:P ratio can reflect the characteristics of the supply and limitation of nitrogen and phosphorus nutrients to plants in the ecosystem (Yang et al., 2020). Changes and the distribution of soil nutrients directly affect the availability of plant nutrients Plant biomass is an important indicator of plant growth (Huang, 2020). The stability of plant elements in different growth and development stages, along with the relationship between plant growth biomass allocation and the content and ratio of nitrogen and phosphorus elements have become the focus of ecological stoichiometry research (Wang and Yu, 2008; Wu et al., 2010). To date, many studies on modeling individual tree biomass in China have been published (Zou et al., 2015). Stands of different ages require different allometric biomass equations (Mensah et al., 2016). Because of the differences in studies (Wang et al., 2013; Lie and Xue, 2016; Yang et al., 2019), there are significant differences in tree biomass among different age groups, leaving some researchers believe that plants with stable stoichiometric characteristics have higher and more stable biomass (Güxsewell and Gessner, 2009). Biomass is the material and energy basis of the entire forest ecosystem and is the basis for studying the productivity, net primary productivity, and carbon cycle in a forest. Metrological characteristics can intuitively reflect plant nutrient content and nutrient utilization strategies (Li et al., 2015). The distribution pattern and changes in the nutrient content of the soil will greatly affect the growth and development of plants in the forest stand (Liu et al., 2010). Therefore, understanding the terrestrial ecosystem which includes plant leaves and soil carbon, nitrogen, and phosphorus nutrient elements and their relationship with biomass, along with exploring the sources of plant nutrients in the soil nutrient cycling and balance constraint relationship between them, has important significance in improving the refinement of local forestry management.

At present, ecological stoichiometry has made great progress worldwide, especially in the study of forest and aquatic ecosystems. Compared with Western countries, China’s relevant research theory has had a late start, and there is still a big gap within the knowledge. Since Elser et al. (1996) first clearly put forward the concept of ecological stoichiometry, an increasing number of ecologists have begun to pay attention to and devote themselves to this field (Elser et al., 2000a; Elser et al., 2000b; Tessier and Raynal, 2003; Güsewell, 2004; Sistla and Schimel, 2012). Some researchers have studied the stoichiometric characteristics of plant leaves, other organs, and soil. And one of the studies found that the nitrogen content in the leaves of the three main plants in the Songnen plain had great differences, while the phosphorus content showed no significant differences. The N:P ratio in plant leaves was greatly affected by the content of soil nutrients, such as carbon, nitrogen, and phosphorus. Studies have shown that leaf nitrogen and phosphorus content can affect the final allocation of plant biomass (Usuda, 1995; Andrews et al., 2006; Kerkhoff and Enquist, 2006; Ren et al., 2007; Fan et al., 2008; Ding et al., 2011; Xu and Jiang, 2015). It indicated that the nutrient content of soil had certain influence on the nutrient content and biomass of leaves. Forest ages and site conditions are important factors for studying plant and soil nutrients. There are few studies on plant-soil nutrients including forest ages and site conditions. Therefore, it is very necessary and important to study the effects of soil carbon, nitrogen and phosphorus on leaf biomass and carbon, nitrogen and phosphorus contents of leaves under different forest ages and site conditions.

*Pinus tabuliformis* is an important tree species for afforestation and soil and water conservation in the temperate mountainous area of China and a large area of *P. tabuliformis* plantations has been formed, which plays a very important ecological role in the environment of the area. In our previous study found a conclusion that the carbon content of the leaves and the TC, TN, and TP content of the soil of *Pinus tabuliformis* plantations in the temperate mountainous area of China changed with forest ages and the accumulations of soil TC, TN, and AP are long-term process, and TP content has the most evident accumulation trend with an increase in forest ages (Wang et al., 2021). Therefore, based on the previous research results, this study was aimed to analyze leaf biomass and the changes of leaf total carbon (LTC), total nitrogen (LTN), total phosphorus (LTP), and soil total carbon (STC), total nitrogen (STN), total phosphorus (STP), available phosphorus (SAP) in *P. tabuliformis* plantations over the entire life cycle under different site conditions in a more in-depth manner. The purpose of this study was to determine:1) the changes of leaf biomass and element content in *P. tabuliformis* plantations at different forest ages and site conditions; 2) effects of soil C: N: P stoichiometry on leaf biomass and leaf C: N: P stoichiometry.

## 2 Materials and methods

### 2.1 Overview of the study area

The sample plots are located in Fushun County, a mountainous area of eastern Liaoning Province in the temperate humid climate region of China, which belongs to the temperate monsoon climate zone with cold winters and rainy summers. The average annual precipitation is 700–850 mm, average annual temperature is 4–11°C, the soil is mainly dark brown loess, generally acidic or neutral loess. *Quercus mongolica*, *Juglans mandshurica*, *Rubus crataegifolius*, *Alnus sibirica*, *Lespedeza bicolor*, and *Corylus heterophylla* were the main understory plants.

Fixed sample plots of *Pinus tabuliformis* plantations are located in Magu Forest Farm of Fushun Mining Group Co., Ltd. in the eastern mountainous area of Liaoning Province. A total of 30 sample plots were set up for the study, each with an area of 0.06 hm^2^ (20 m × 30 m)。Fifteen of the sample plots had a low mountain sunny slope and the other 15 plots had a low mountain shady slope. The stands of each site condition included five age classes: young stage (YS ≤ 20a), half mature stage (20a<HMS ≤ 30a), near mature stage (30a<NMS ≤ 40a), mature stage (40a<MS ≤ 60a) and over mature stage (OMS>60a). In each sample plot, two standard trees were selected and their height and diameter at breast height were measured, and stand density, stand age, stand average diameter at breast height, stand average height and other stand indicators were also investigated. The profiles of the sample plots are listed in **Table 1**.

**Table 1:** Profiles of the sample plots.

Age class	Mean DBH (cm)		Mean tree height (m)		Mean stand density(tree·hm^-2^)	
	S1	S2	S1	S2	S1	S2
YS	6.3 ± 0.9	7.8 ± 1.2	3.8 ± 0.5	4.5 ± 0.3	1600 ± 52	1600 ± 67
HMS	18.0 ± 0.6	16.6 ± 1.1	10.7 ± 0.6	12.9 ± 1.3	1255 ± 31	1255 ± 35
NMS	20.3 ± 1.3	20.8 ± 1.3	12.3 ± 1.2	13.0 ± 0.8	1089 ± 118	1083 ± 108
MS	20.7 ± 2.2	23.5 ± 4.7	12.6 ± 2.0	13.2 ± 0.5	900 ± 106	900 ± 100
OMS	27.5 ± 1.2	26.4 ± 1.8	15.1 ± 2.9	13.3 ± 1.1	563 ± 108	563 ± 98

S1, Sunny slope; S2, Shady slope.

### 2.2 Leaf samples

Two standard branches were selected from each of the three layers (upper, middle, lower) of the canopy for needle leaf sampling in October 2019. Needle leaf samples collected from each standard branch were mixed and stored in an envelope. Six needle leaf samples were collected from each standard tree crown, and a total of 360 needle leaf samples were collected from all 30 sample plots. Needle leaf samples were weighed at the sample plots for fresh weight and brought back to the laboratory. Fresh leaf samples were killed using an oven at 105°C and dried at 65°C to a constant weight, then weighed for dry weight and the biomass of the needle leaves was calculated.

### 2.3 Determination of biomass of leaf per plant

In order to reduce the error caused by water loss of needles during the removal of needles from standard branches, the fresh weight of each layer of standard branches with leaves was weighed and recorded as M_1_, and the fresh weight of branches was recorded as M_2_ after the removal of the fresh leaves, and the fresh weight of needles was recorded as M_1_-M_2_. After weighing the fresh weight of the sampling branches and sample leaves, they were placed in a laboratory oven and baked to a constant weight at a constant temperature of 80°C. Then, the biomass calculation formula for each layer of leaves is as follows:


$$\text{W}=\left({\text{M}}_{1}-{\text{M}}_{2}\right)\times \text{N}\times \text{P}$$

where W is leaf biomass, M_1_ is the standard branch weight with leaves, M_2_ is the standard branch weight, N is the number of standard branches, and P is the leaf water content (dry weight of sample leaves versus fresh weight of sample leaves).

The obtained leaf biomass of each layer was added as the biomass of leaf per plant.

### 2.4 Soil samples

In October 2019, a soil pit (1m× 0.5m × 0.6m) was excavated at 0.5m and 1m away from the standard tree trunk in each sample plot. Set three soil layers (0-20 cm, 20-40 cm, and 40-60 cm) in the soil pit, and collect soil mixed samples of each layer. A total of 360 soil samples were collected. Soil samples were brought back to the laboratory, dried and subjected to soil nutrient measurements.

### 2.5 Elements in leaf and soil samples

Needle samples were screened using a 60-mesh sieve and soil samples were screened using a 100-mesh sieve. Appropriate samples were weighed to determine the nutrients of needles and soil. Total carbon and total nitrogen contents of soil and needle were measured using an elemental analyzer (Vario EL III, Elementa Langensel bold, Germany). Total phosphorus content of needles is the molybdenum blue colorimetric-spectrophotometer method. Total phosphorus in soil was determined by the HCLO_4_-H_2_SO_4_-molybdenum-antimony colorimetric method, and available phosphorus in soil was determined by the molybdenum-antimony colorimetric method (Zhang J, 2019).

### 2.6 Statistical analysis

Data were analyzed using SPSS 22.0 software (SPSS, Inc., Chicago, IL, USA). The differences in the biomass, total carbon, total nitrogen, and total phosphorus content in leaf and soil ecological stoichiometry and its ratio throughout different age classes in entire life cycle at different site conditions were examined using single factor variance analysis. Duncan’s multiple comparison method was used for significance analysis (*p*< 0.05). Furthermore, the ecological stoichiometry and ratio of soil and its correlation with leaf biomass was also analyzed by Person correlation analysis. The same correlation analysis was used to examine the ecological stoichiometry and ratio of soil and its correlation with leaf biomass. The relationships between the ecological stoichiometry and the ratio of the surface soil (0-20 cm), the ecological stoichiometry and ratio of leaves, along with the ecological stoichiometry and ratio of soil and its correlation with leaf biomass in *P. tabuliformis* plantations were analyzed by Pearson correlation analysis.

## 3 Results

### 3.1 Leaf biomass per plant in *Pinus tabuliformis* plantation

**Figure 1:**
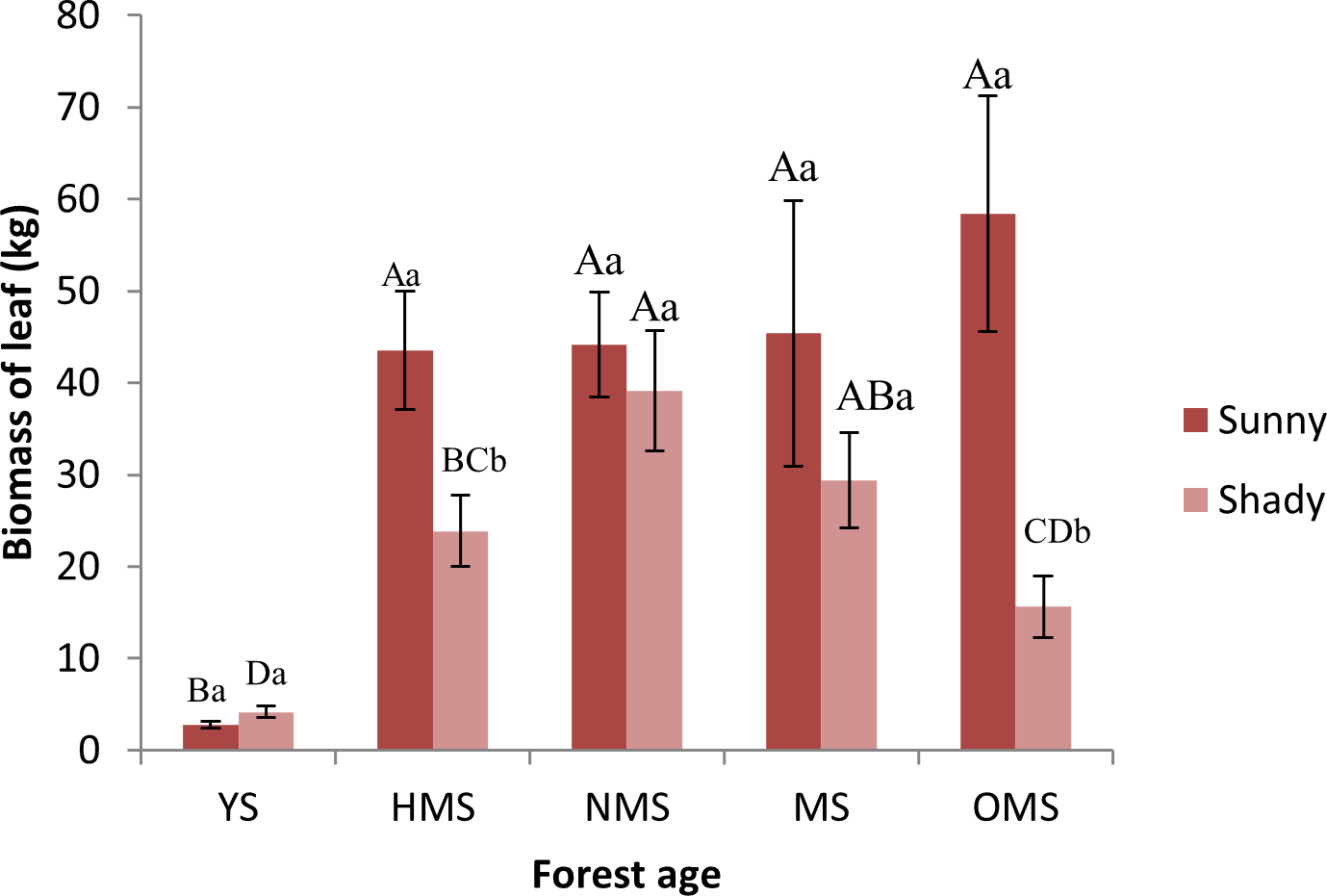
Biomass of leaf based on forest ages and site conditions. Different capital letters indicate significant differences in forest ages, and different lowercase letters indicate significant differences sites conditions (*p<* 0.05). YS, Young stage; HMS, Half-mature stage; NMS; Near-mature stage; MS, Mature stage; OMS, Over-mature stage; Sunny, Sunny slope; Shady, Shady slope.

Over the entire life cycle of the *P. tabuliformis* plantation, the average biomass of leaves per plant was 38.86 kg on sunny slopes and 22.43 kg on shady slopes. The leaf biomass of the sunny slope gradually increased with increasing forest ages and reached a maximum in the OMS (**Figure 1**). The leaf biomass of YS was significantly different from that of the other forest ages. The leaf biomass of the shady slope first increased and then decreased with the increase in forest ages and reached the maximum value in NMS. At the same forest ages, the biomass of leaves at different site conditions was significantly different only in HMS and OMS. And the biomass of leaves on the sunny slope was higher than that on the shady slope.

### 3.2 Leaf ecological stoichiometric characteristics

#### 3.2.1 LTC, LTN, and LTP

**Figure 2:**
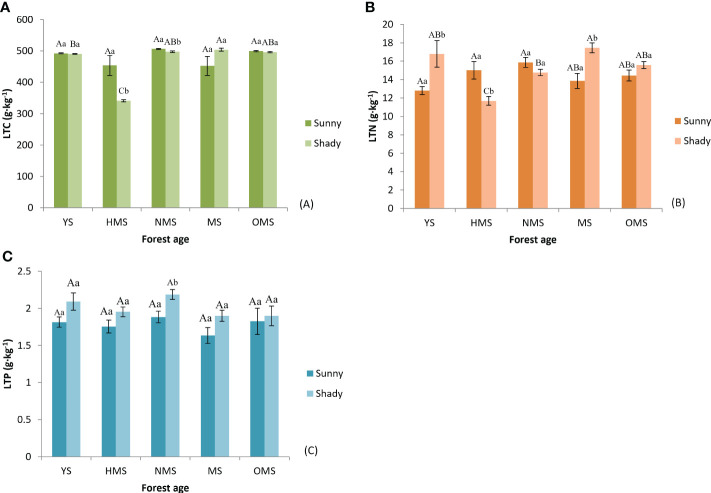
The total carbon of leaf **(A)**, total nitrogen of leaf **(B)**, and total phosphorus of leaf **(C)**. Different capital letters indicate significant differences in forest ages, and different lowercase letters indicate significant differences sites conditions (*p<* 0.05). YS, Young stage; HMS, Half-mature stage; NMS, Near-mature stage; MS, Mature stage; OMS, Over-mature stage; Sunny, Sunny slope; Shady, Shady slope.

Over the entire life cycle of the *P. tabuliformis* plantation, the average total carbon in the leaves was 480.89 g·kg^-1^ on sunny slopes and 465.86 g·kg^-1^ on shady slopes (**Figure 2A**); the average total nitrogen in the leaves was 14.39 g·kg^-1^ on sunny slopes and 15.25 g·kg^-1^ on shady slopes (**Figure 2B**), and the average total phosphorus in the leaves was 1.78 g·kg^-1^ on sunny slopes and 2.01 g·kg^-1^ on shady slopes (**Figure 2C**).

In the same site conditions, there was no significant difference in the total carbon, total nitrogen, and total phosphorus in the leaves of sunny slopes among different ages. The total carbon of leaves in shady slopes was significantly different between the other forest ages, excluding NMS and OMS. Except for YS and OMS, there was a significant difference in the total nitrogen of leaves between the other forest ages. There was no significant difference in the total phosphorus of leaves among the different forest ages. In the same forest age, the total carbon of leaves in HMS and NMS was significantly different between the two site conditions. The total nitrogen of leaves in YS, HMS, and MS was significantly different between the two site conditions while the total phosphorus of leaves in NMS was significantly different between the two site conditions. The average total nitrogen and total phosphorus of leaves on shady slopes were higher than those on sunny slopes, but the regular of total carbon of leaves was the opposite.

#### 3.2.2 Stoichiometric ratio of LTC, LTN, and LTP

**Figure 3:**
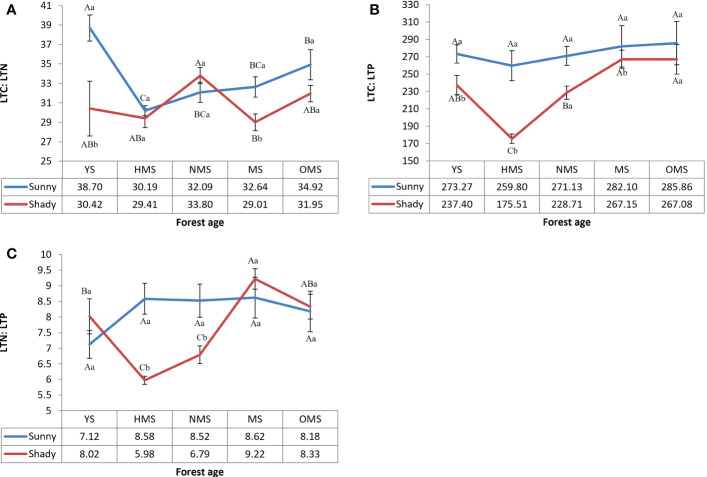
LTC : LTN of leaves **(A)**, LTC : LTP of leaves **(B)**, and LTN : LTP of leaves **(C)**. Different capital letters indicate significant differences in forest ages, and different lowercase letters indicate significant differences sites conditions (*p<* 0.05). YS, Young stage; HMS, Half-mature stage; NMS, Near-mature stage; MS, Mature stage; OMS, Over-mature stage. Sunny, Sunny slope; Shady, Shady slope.

Over the entire life cycle of the *P. tabuliformis* plantation, the average LTC : LTN ratio was 33.71 in the sunny slope and 30.92 in the shady slope (**Figure 3A**), the average LTC : LTP ratio was 274.43 in the sunny slope and 235.17 in the shady slope (**Figure 3B**), and the average LTN : LTP ratio was 8.21 in sunny slopes and 7.67 in shady slopes (**Figure 3C**). The results showed that LTC : LTN had no obvious change with forest ages. LTC : LTP and LTN : LTP both reached their maximum values at MS or OMS. LTC : LTN, LTC : LTP, and LTN : LTP of sunny slopes was higher than that of shady slopes.

### 3.3 Soil ecological stoichiometric characteristics

#### 3.3.1 STC, STN, STP, and SAP

**Figure 4:**
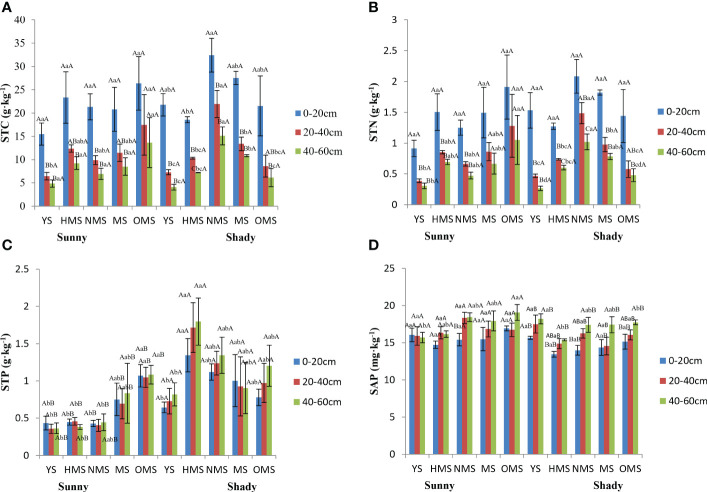
STC of soil **(A)**, STN of soil **(B)**, STP of soil **(C)**, and SAP of soil **(D)**. Different capital letters indicate significant differences in soil layers and different lowercase letters indicate significant differences forest ages, and additional different capital letters indicate significant differences in site conditions (*p<* 0.05). YS, Young stage; HMS, Half-mature stage; NMS, Near-mature stage; MS, Mature stage, OMS, Over-mature stage. Sunny: Sunny slope, Shady: Shady slope.

Over the entire life cycle of the *P. tabuliformis* plantation, the average STC was between 4.88 and 26.34 g·kg^-1^ in sunny slopes and in shady slopes it ranged between 4.09 and 32.42 g·kg^-1^ (**Figure 4A**), while the average STN was between 0.31 and 1.91 g·kg^-1^ in sunny slopes and in shady slopes it was between 0.27 and 2.08 g·kg^-1^ (**Figure 4B**). The average STP ranged from 0.36 to 1.08 g·kg^-1^ in sunny slopes and in shady slopes it was between 0.64 and 1.80 g·kg^-1^ (**Figure 4C**), and the average SAP was 14.72 to 19.06 mg·kg^-1^ in sunny slopes and between 13.97 and 18.20 mg·kg^-1^ in shady slopes (**Figure 4D**).

The STC and STN were not significantly different under different site conditions, but the STP and SAP were significant differences under different site conditions. The order of the STC and STN in different soil layers were 0-20 cm, 20–40 cm, 40-60 cm. Moreover, the topsoil content (0–20 cm) was significantly different from that of the other soil layers. However, the STP and SAP in different soil layers were generally found to be greater in the following order of soil depth: 40–60 cm, 20–40 cm, 0–20 cm. The STC and STN decreased with increasing soil depth, while the STP and SAP were opposite. There was no significant difference in the STP among the different soil layers. However, there was a significant difference in the SAP between the 40cm and 60 cm soil layer and other soil layers. In the sunny slopes, the element contents did not change with forest ages, but almost all reached the maximum value in the OMS. In shady slopes, the STC, STN and STP in different soil layers changed with forest ages in the same way, both increased first and then decreased. The SAP in different soil layers decreased first and then increased with the increase in forest ages, and the maximum value was found in YS.

#### 3.3.2 Stoichiometric ratio of STC, STN, and STP

**Figure 5:**
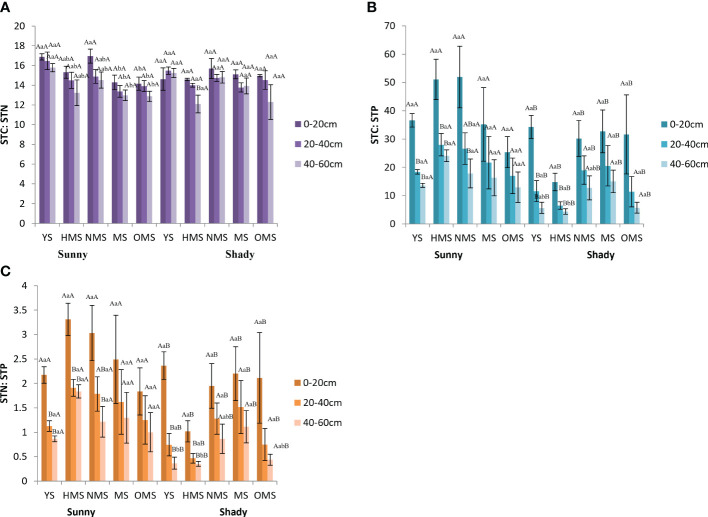
STC : STN **(A)**, STC : STP **(B)**, and STN : STP **(C)**. Different capital letters indicate significant differences in soil layers and different lowercase letters indicate significant differences forest ages, and additional different capital letters indicate significant differences in site conditions (*p<* 0.05). YS, Young stage; HMS, Half-mature stage; NMS, Near-mature stage; MS, Mature stage; OMS, Over-mature stage. Sunny: Sunny slope, Shady: Shady slope.

Over the entire life cycle of the *P. tabuliformis* plantation, the average STC : STN was between 12.87 and 16.94 in sunny slopes and in the shady slopes it ranged from 13.97 to 18.20 (**Figure 5A**). The average STC : STP was found to be between 12.97 and 51.93 in sunny slopes and in shady slopes it was between 4.37 and 34.26 (**Figure 5B**), while the average STN : STP ranged from 0.87 to 3.31 in sunny slopes and 0.35 and 2.36 in shady slope (**Figure 5C**).

STC : STN showed no significant difference under different site conditions, while STC : STP and STN : STP were significantly different under different site conditions. On sunny slopes, STC : STN, STC : STP, and STN : STP showed a downward trend with increasing soil depth. STC : STP and STN : STP first increased and then decreased with increasing forest ages. In shady slopes, STC : STN changed irregularly with soil depth, and both STC : STP and STN : STP showed a decreasing trend with increasing soil depth.

### 3.4 Correlation of leaf ecological stoichiometry and biomass with soil ecological stoichiometry

As shown in **Table 2**, in the sunny slopes, in YS, STC : STN had a significant negative correlation with LTN and LTN : LTP, and a significant positive correlation with LTC : LTN. STN : STP was significantly and positively correlated with LTC. There was a significant positive correlation between LTN and LTP. LTC : LTN, LTC : LTP, and LTN : LTP were significantly negatively correlated. In HMS, there was a significant negative correlation between biomass and LTN, and LTP and LTC : LTP was significantly negatively correlated. In NMS, there was a significant negative correlation between biomass and LTC : LTP. In MS, STC was negatively correlated with LTP and positively correlated with LTC : LTP. STN and LTC : LTP showed a significant positive correlation. SAP was positively correlated with LTC. LTC and LTN : LTP showed a significant negative correlation. In the OMS, SAP had a significant positive correlation with LTC : LTN. There was a significant positive correlation between biomass and STN : STP. LTP and LTN : LTP showed a significant negative correlation.

**Table 2:** Correlations of leaf biomass and ecological stoichiometry with soil ecological stoichiometry.

Site	Age	variable	Biomass	LTC	LTN	LTP	LTC : LTN	LTC : LTP	LTN : LTP
Sunny slope	YS	STC : STN	-0.594	-0.762	-.998*	0.982	.999*	-0.995	-1.000**
		STN : STP	-0.13	.998*	0.764	-0.575	-0.696	0.644	0.73
		LTN	0.54	0.803	1	-0.967	-0.995	0.986	.999*
		LTC : LTN	-0.621	-0.74	-0.995	0.988	1	-.998*	-.999*
	HMS	Biomass	1	-0.86	-.998*	0.653	0.982	-0.677	-0.814
		LTP	0.653	-0.175	-0.604	1	0.497	-1.000*	-0.972
	NMS	Biomass	1	0.966	-0.556	0.994	0.463	-.997*	-0.797
	MS	STC	0.423	-0.847	0.214	-.998*	-0.096	.998*	0.869
		STN	0.298	-0.768	0.081	-0.981	0.038	.997*	0.795
		SAP	-0.871	.998*	-0.742	0.846	0.657	-0.775	-0.995
		LTC	-0.84	1	-0.7	0.877	0.61	-0.812	-.999*
	OMS	SAP	-0.371	-0.966	-0.987	-0.877	1.000*	0.917	0.873
		STN : STP	1.000*	0.579	0.494	-0.146	-0.374	0.053	0.153
		LTP	-0.122	0.722	0.788	1	-0.863	-0.996	-1.000**
Shady slope	YS	LTC	-0.567	1	.999*	0.986	-.999*	-0.979	0.984
		LTN	-0.606	.999*	1	0.977	-1.000**	-0.968	0.992
		LTP	-0.423	0.986	0.977	1	-0.978	-.999*	0.941
	NMS	Biomass	1	.999*	-0.012	-0.727	0.303	0.825	0.498
		STP	0.858	0.88	0.504	-0.976	-0.23	.998*	0.873
		STC : STP	-0.831	-0.856	-0.546	0.986	0.278	-1.000**	-0.896
		STN : STP	-0.675	-0.708	-0.73	.997*	0.499	-0.974	-0.976
	MS	STC : STN	0.312	-0.619	0.487	-0.675	-0.49	0.551	.999*
		STN : STP	-0.333	0.637	-0.506	0.658	0.51	-0.532	-.998*
		LTN	0.982	-0.987	1	0.316	-1.000**	-0.461	0.454
	OMS	STC : STP	.997*	-0.887	0.882	0.927	-0.874	-0.891	-0.911
		LTC	-0.85	1	-1.000**	-0.996	1.000*	1.000**	.999*
		LTN	0.845	-1.000**	1	0.995	-1.000*	-1.000*	-.998*
		LTP	0.896	-0.996	0.995	1	-0.993	-0.996	-.999*

*p < 0.05, **p < 0.01. Only the results with significant correlations are presented in the table. YS, Young stage; HMS, Half-mature stage; NMS, Near-mature stage; MS, Mature stage; OMS, Over-mature stage. LTC, Leaf total carbon; LTN, Leaf total nitrogen; LTP, Leaf total phosphorus; STC, Soil total carbon; STN, Soil total nitrogen; STP, Soil total phosphorous; SAP, Soil available phosphorous; Site, site factors, including shady slope and sunny slope. Age: stand age class Variable: all factors for correlation comparison. Biomass: leaf biomass per plant; LTC, Leaf total carbon; LTN, Leaf total nitrogen; LTP, Leaf total phosphorus. The meaning of bold value indicates the significant correlation between leaf biomass, leaf ecological stoichiometry and soil ecological stoichiometry under different forest ages and site conditions.

In the shady slopes, there is a significant positive correlation between LTC and LTN in YS, and a significant negative correlation between LTC, LTN, and LTC. LTN and LTC : LTN were negatively correlated. LTP and LTC : LTP showed a significant negative correlation. In NMS, there was a significant positive correlation between biomass and LTC. STP and LTC : LTP showed a significant positive correlation. STC : STP and LTC : LTP were significantly negatively correlated. STN : STP and LTP showed significant positive correlations. In MS, STC : STN, and LTN : LTP showed a significant positive correlation, while STN : STP and LTN : LTP showed a significant negative correlation. The correlation between LTN and LTC LTN was significantly negative. In the OMS, there was a significant positive correlation between biomass and STC : STP, while there was a significant negative correlation between LTC and LTN, a significant positive correlation between LTC and LTN, a significant positive correlation between LTC and LTP, and a significant positive correlation between LTC : LTP and LTN : LTP. There were significant negative correlations between LTN and LTC : LTN, LTC : LTP, and LTN : LTP. LTC : LTN, and LTC : LTP, LTC : LTP, and LTN : LTP were significantly positively correlated, while LTN : LTP and LTP showed a significant negative correlation

## 4 Discussion

### 4.1 Effects of site conditions and forest ages on leaf biomass

In this study, the result (**Figure 1**) is consistent with the results of Huang (2020) and Zhang P (2019). *P. tabuliformis* is a coniferous evergreen tree of *Pinaceae*, The species is greatly affected by light and because light had a certain promotion effect on the increase in leaf biomass, the leaf biomass of sunny slopes was slightly higher than that of shady slopes. And with an increase in forest ages, the leaf biomass showed an increase with the increase in forest ages. At the later stage of tree growth, the biomass growth of each stand stage showed a decreasing trend, indicating that the growth of trees was gradually stable, especially at the aged stage. Some studies believe that the death of trees is one of the main reasons for the decrease in forest biomass in the old forest age stage (Xu et al., 2012). In addition, the light is not sufficient on the shady slope, therefore the leaf biomass of *P. tabuliformis* may decrease after it enters the aging stage. These results determined the changes of leaf biomass and element content in *P. tabuliformis* plantations under different forest ages and site conditions.

### 4.2 Effects of site conditions and forest ages on leaves and soil C:N:P stoichiometric characteristics

In this study, the results (**Figures 2A, 2B and 2C**) which was inconsistent with previous research results (Liu et al., 2015; Wang et al., 2015; Jiang et al., 2016). These results may be influenced by different factors such as sampling time, forest ages, site conditions, and climate environment of the growing place. This study also found that because of the fact that the *P. tabuliformis* plantation with NMS was in the peak growth period and may needed more rRNA to meet the protein synthesis, thus leading to an increase in leaf nitrogen content. In shady slopes, leaf total nitrogen of the *P. tabuliformis* plantation reached the maximum value in MS, which may be due to a lack of light. In addition, this species was in the peak growth stage in the MS stage. The change of total nitrogen in *P. tabuliformis* needles between forest ages directly affected the difference between forest ages of LTC : LTN and LTC : LTP. The changes in total carbon, total nitrogen, and total phosphorus of the needles of the five forest ages were not consistent, which might be because the absorption and demand of soil nutrients were different with the increase in the age of the forest. In addition, the supply of soil nutrients under the forest also changed with the change of time and the comprehensive influence of various factors (Wang et al., 2011). The total carbon of the leaves on the sunny slopes was higher than that of the leaves on the shady slopes, which may be because the leaves on sunny slopes undergo photosynthesis and accumulate more nutrients than those on shady slopes. The results verified that the element content of *P. tabuliformis* plantation leaves were related to forest ages and site conditions. The leaf total carbon of *P. tabuliformis* was significantly higher than that of 492 other terrestrial plants (464 g·kg^-1^) studied by Elser et al. (2000a), indicating that the leaf organic compound content of *P. tabuliformis* was higher.

The total nitrogen of leaves was significantly lower than the average nitrogen content of Chinese plants (20.2 g·kg^-1^) and the average nitrogen content of global plants (20.6 g·kg^-1^). In contrast, the total phosphorus in leaves was slightly higher than the average phosphorus content in China (1.46 g·kg^-1^) and the average phosphorus content of global plants (1.99 g·kg^-1^). The results showed that there was a lack of nitrogen in *P. tabuliformis*. Some studies have shown that when LTN : LTP is >16, plant growth is restricted by phosphorus. When LTN : LTP was less than 14, plant growth is considered to be restricted by nitrogen and when LTN : LTP is between 14 and 16, plant growth is considered to be restricted by both (Koerselman and Meuleman, 1996). In this study, the result (**Figure 3C**) showed that the growth of *P. tabuliformis* plantations was mainly restricted by nitrogen. In the management of plantations, especially in the young stage of *P. tabuliformis* plantations, nitrogen fertilizer should be reasonably applied to improve the soil nutrient supply. Based on the principle of LTN : LTP stoichiometry, the changes in the LTN : LTP ratio in different age communities were studied. It was found that the main nutrient elements limiting plant growth in different age groups are of great significance for forest management to improve productivity.

The results of soil stoichiometric characteristics are consistent with the research results of Zhao et al. (2012). The reason may be that woodland litter gradually increases with an increase in time, so soil elements are accumulating. However, in shady slopes, it may be that in the late growth period, due to insufficient illumination, weakened photosynthesis, and reduced synthetic organic matter, the demand of plants for soil nutrients increased, so the nutrient content in the soil decreased. The STC and STN were the highest in the surface layer (0–20 cm), showing a “surface aggregation” phenomenon, which was consistent with previous research results (Pan et al., 2011). In this study, the STP was lower than the global average level (2.8 g·kg^-1^) (Ren et al., 2007), which is consistent with the previous findings reporting that soil phosphorus content in China is generally lower than the global level (Yang et al., 2014). Soil phosphorus is made available from the differentiation of soil minerals and the activities of microorganisms, and with an increase in time, the external environment changes, and the hydrothermal conditions also change, leading to a change in phosphorus content. However, the main source of available phosphorus in soil is rock and mineral weathering, which is a steady and lengthy process. Therefore, soil phosphorous did not significantly change due to soil depth. In this study, because the growth of *P. tabuliformis* in sunny slopes is better than that in shady slopes, and the nutrient demand is higher. Therefore, the soil transports more nutrients to *P. tabuliformis* in sunny slopes, so the nutrient content in the soil is relatively low.

### 4.3 C:N:P stoichiometric characteristics of soil and leaves

In this study, based on the correlation between leaf biomass and leaf total carbon, total nitrogen and total phosphorus, and soil total carbon, total nitrogen, and total phosphorus, it can be concluded that leaf biomass was related to leaf total carbon, total nitrogen, and the LTC : LTP ratio, and STC : STP and STN : STP. In other words, leaf biomass can be adjusted by adjusting the soil total carbon, total nitrogen, and total phosphorus. When soil total nitrogen is sufficient, the leaf biomass can be improved. The results confirmed that soil C:N:P stoichiometric characteristics have a certain influence on leaf biomass. The stoichiometric characteristics of nitrogen and phosphorous in plant leaves were not significantly correlated with biomass change. This result is consistent with the findings of Yan et al. (2015). In shady slopes and YS, it may be that the plants are in the early stage of structure construction and growth, which requires a large amount of protein synthesis from nitrogen and is also the period of biomass accumulation. Therefore, there is a significant positive correlation between LTC and LTN. In the OMS, LTC and LTN were negatively correlated. This result is consistent with the results of Dong (2017), which may be due to the contradictory allocation of leaf nutrients between structural construction and rapid plant growth.

Under different site conditions, according to the correlation between leaf total carbon, total nitrogen, and total phosphorus and soil total carbon, total nitrogen, and total phosphorus, it can be seen that the leaf carbon, nitrogen, and phosphorus content can be adjusted by adjusting the soil carbon, nitrogen, and phosphorus content. This result confirmed that soil C:N:P stoichiometric characteristics significantly affect leaf C:N:P stoichiometric characteristics.

## 5 Conclusions

The forest ages and site conditions of *P. tabuliformis* plantations had significant effects on leaf biomass and site conditions had a certain effect on the total carbon, total nitrogen, and total phosphorus in leaves and soil. Due to light, the biomass, LTC and element ratios of *P. tabuliformis* on sunny slopes were all higher than those on shady slopes. The STC and STN decreased with increasing soil depth. In addition to SAP, the average content of STC, STN, and STP in shady slopes was higher than that in sunny slopes, and the ratios showed the opposite. The results showed that the biomass of leaves was mainly limited by nitrogen, and there was a lack of nitrogen in the leaves. In the management of *P. tabuliformis* plantations in this temperate mountainous area, it is suggested to plant on sunny slopes, and especially during the young stage of *P. tabuliformis* plantations. In addition, it is suggested to apply a reasonable amount of nitrogen fertilizer to improve the soil nutrient supply and increase the biomass of leaves. These results can provide a reference for the management of *P. tabuliformis* plantations in the temperate mountainous area of China.

## Data availability statement

The raw data supporting the conclusions of this article will be made available by the authors, without undue reservation.

## Author contributions

All authors contributed to the article and approved the submitted version.
